# Robot-assisted thoracoscopic resection of a posterior mediastinal tumor with immunoglobulin G4-related disease: a case report

**DOI:** 10.1186/s13019-024-02655-5

**Published:** 2024-05-16

**Authors:** Taihei Takeuchi, Hiromitsu Takizawa, Yoshimi Bando, Akio Hosokawa, Hiroyuki Sumitomo, Naoki Miyamoto, Shinichi Sakamoto, Atsushi Morishita, Naoya Kawakita, Hiroaki Toba

**Affiliations:** 1 Department of Thoracic, Endocrine Surgery, and Oncology, Tokushima University Graduate School of Biomedical Sciences, Tokushima City, Tokushima, 770-8503 Japan; 2 Division of Pathology, Tokushima University Hospital, Tokushima City, Tokushima, 770-8503 Japan

**Keywords:** Immunoglobulin G4-related disease, Mediastinal neoplasms, Robotic surgical procedures

## Abstract

**Background:**

Immunoglobulin (Ig)G4-related disease affects nearly every organ, and its clinical course varies depending on the involved organ; however, its occurrence in the mediastinum is rarely reported.

**Case presentation:**

A 58-year-old woman presented with a posterior mediastinal tumor along the thoracic spine on imaging. Based on her elevated serum IgG4 level of 349.7 mg/dL, IgG4-related disease was suspected. Since the tumor was growing and malignancy could not be excluded, surgical resection was performed for definitive diagnosis. Robot-assisted thoracoscopic surgery was performed via the left semipronation and right thoracic approaches. The irregularly-shaped tumor was located on the level of the seventh to ninth thoracic vertebra, along the sympathetic nerve. A malignancy was not excluded based on the appearance of the tumor. The tumor had poor mobility. The sympathetic nerves, intercostal arteries, and veins were also excised. In this case, the articulated forceps, used during the robotic surgery, were useful in achieving complete tumor resection along the vertebral body. The pathological examination revealed IgG4-positive plasma infiltration, which fulfilled the criteria for IgG4-related diseases. The postoperative course was uneventful, and the patient underwent follow-up on an outpatient basis without additional medications.

**Conclusion:**

The clinical presentation of IgG4-related disease varies, based on the involved organs. This case was rare because the mediastinum was involved, and it emphasized the effectiveness of surgical resection.

**Supplementary Information:**

The online version contains supplementary material available at 10.1186/s13019-024-02655-5.

## Background

Immunoglobulin G4-related disease (IgG4-RD) is a systemic autoimmune disorder characterized by enlarged, massive, and thickened lesions, a high serum IgG4 concentration, marked lymphocytic and IgG4-positive plasma infiltration, and tissue fibrosis [1]. It affects nearly every organ, with varying clinical course depending on the involved organ; however, few reports exist on its occurrence in the mediastinum. In rare cases, particularly when the mediastinum is involved, IgG4-RD diagnosis can be difficult. Furthermore, standardized diagnostic and treatment modalities have not yet been established. A surgical biopsy is therefore essential for a definitive diagnosis, especially for growing lesions and for excluding the possibility of malignancy. This study reports a case of robot-assisted thoracoscopic surgery (RATS) for a posterior mediastinal tumor in a patient with IgG4-RD.

## Case presentation

**Fig. 1:**
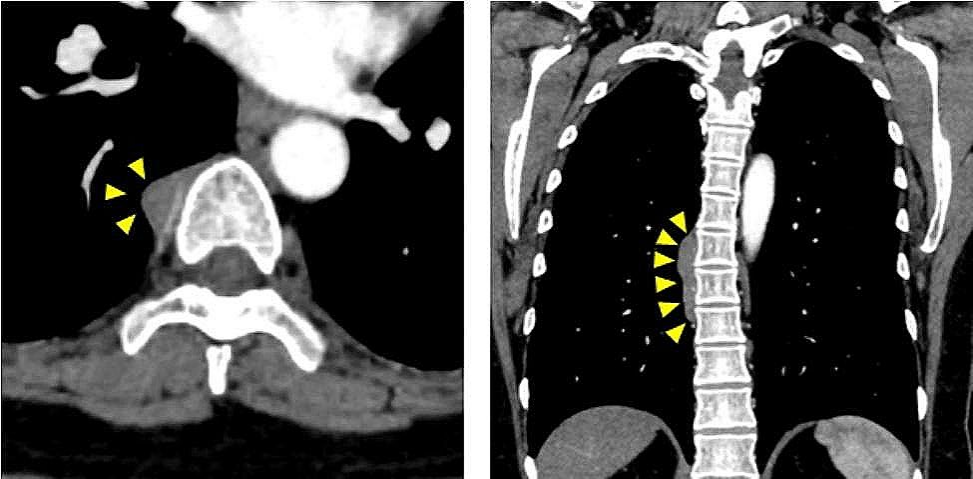
Thoracic contrast-enhanced computed tomography. Thoracic contrast-enhanced computed tomography reveals a mass, measuring 23 × 13 × 55 mm, on the right side of the seventh to ninth thoracic vertebrae. The area indicated by the yellow arrows shows the mass

A 58-year-old woman was referred to our hospital for a detailed examination of a posterior mediastinal tumor. She had been receiving treatment for hypertension and type 2 diabetes mellitus. She underwent surgery for sialadenitis at 40 years of age and developed a pancreatic nodule at 45 years of age; she was being followed up with magnetic resonance imaging (MRI). The patient reported no new subjective symptoms. The MRI, however, revealed a posterior mediastinal tumor along the thoracic vertebral bodies 3 years ago. Over the next 3 years, the tumor size increased by 1 cm. Because the patient had a history of salivary gland inflammation and autoimmune pancreatitis, IgG4-related disease was suspected. Her serum IgG4 concentration was measured preoperatively and was found to be elevated at 349.7 mg/dL. Contrast-enhanced computed tomography showed a smooth tumor, measuring 55 mm, along the right side of the seventh to ninth thoracic vertebrae, with no invasion into the surrounding tissues (Fig. 1). Therefore, an IgG4-related disease affecting the posterior mediastinum was suspected. The tumor grew over time, and a histological diagnosis was required to exclude malignancy. However, performing a needle biopsy was deemed difficult due to the tumor’s location. Thus, complete surgical excision of the tumor was performed.

**Fig. 2:**
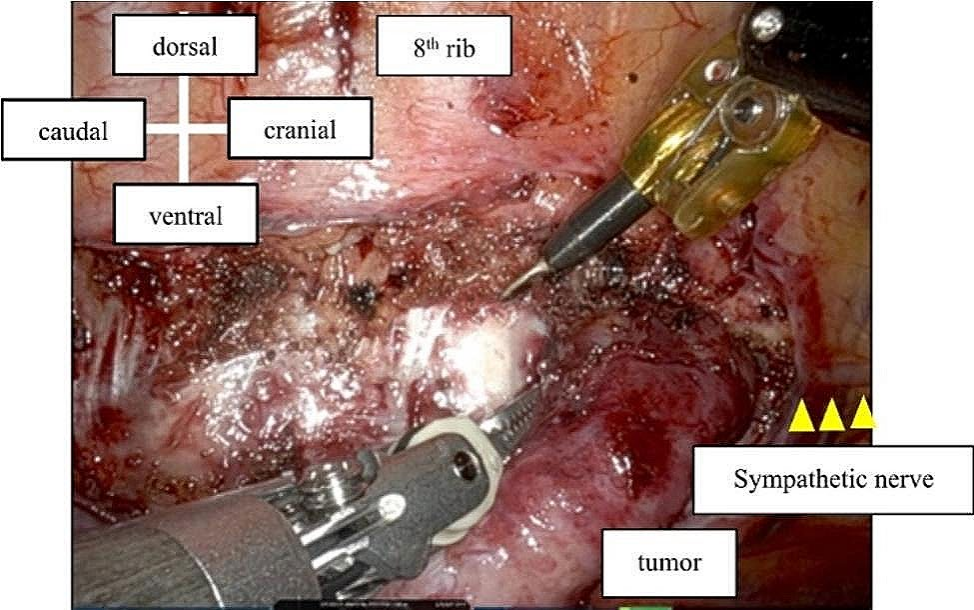
Tumor resection. Complete tumor resection was achieved by robot-assisted thoracic surgery

**Fig. 3:**
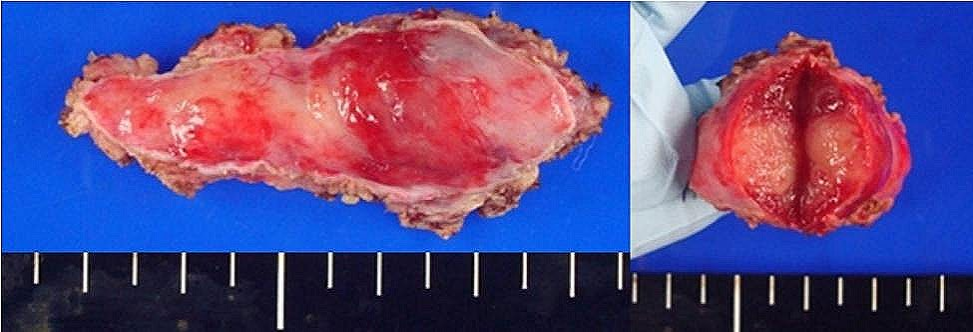
Macroscopic examination. Macroscopic examination reveals elastic, red tumor with thickened soft tissue at the center of the sympathetic nerve

**Fig. 4:**
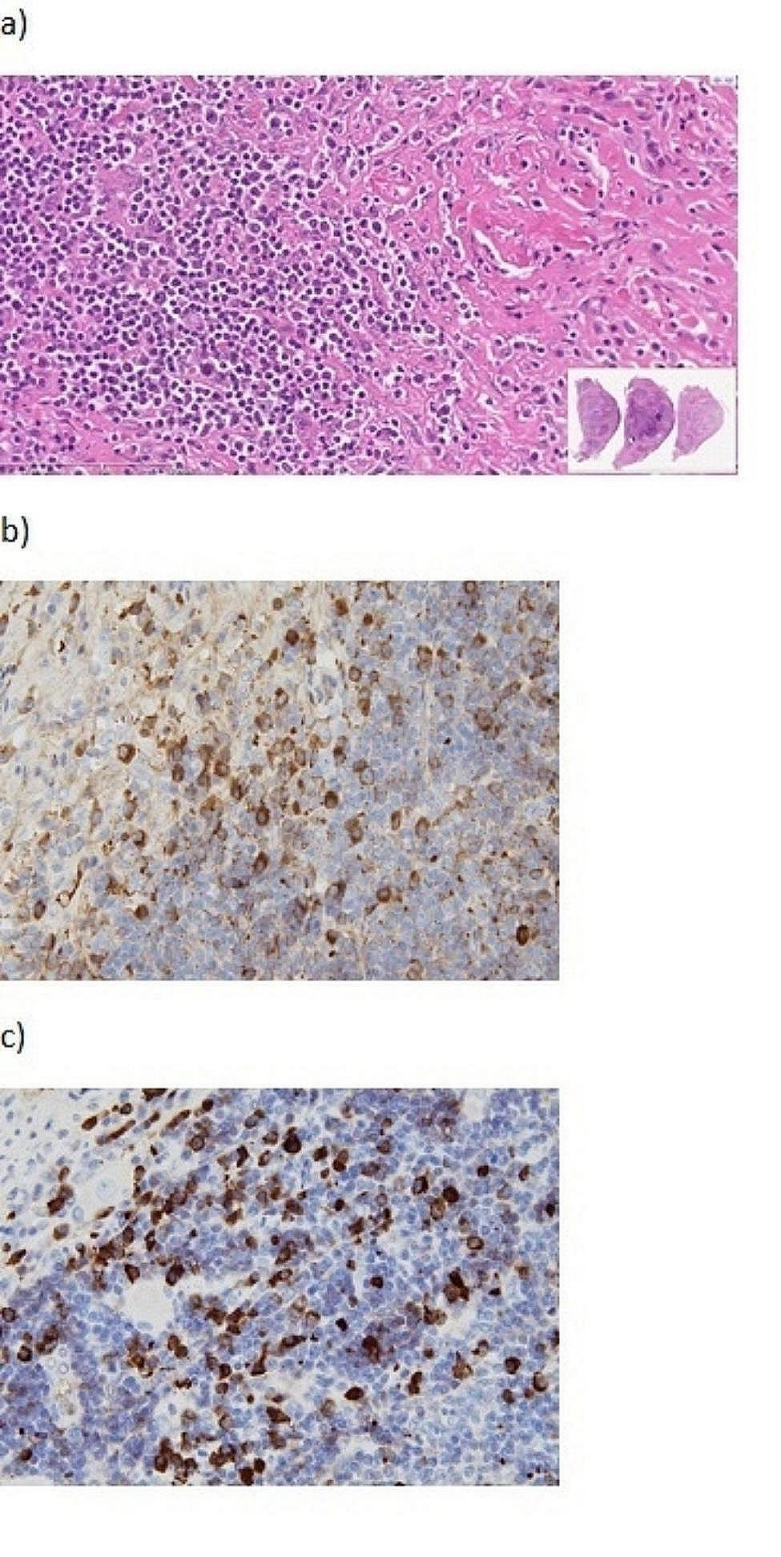
Hematoxylin-eosin and immunohistochemical staining of specimen. (**a**) Hematoxylin-eosin staining of the specimen at (×40). (**b**) Immunoglobulin G immunohistochemical staining (×400). (**c**) Immunoglobulin G4 immunohistochemical staining (×400)

RATS was performed via the right thoracic approach with the patient in a left semi-prone position. During the procedure, carbon dioxide gas was insufflated into the thoracic cavity through a trocar at a pressure of 8 mmHg. Then, 8-mm trocars were inserted into the third, fifth, and eighth intercostal spaces (ICS) through the anterior axillary line for the da Vinci surgical system. A 12-mm trocar was inserted for the assistant at the eighth ICS through the middle axillary line. The irregularly-shaped tumor was located at the level of the seventh to ninth thoracic vertebrae along the sympathetic nerve. It was elastic, hard, and firmly attached to the chest wall. The parietal pleura around the tumor was opened for mobilization. The sympathetic nerve, three intercostal veins, and two intercostal arteries were excised, and the tumor was removed using the joint motion of the robot arm (Figs. 2 and 3). The total duration of the surgery was 139 min. Histological examination revealed infiltration of lymphocytes and plasma cells with collagenous fibers. In the immunohistochemical analysis, a proportion of IgG4-positive cells/IgG-positive cells exceeding 60% was observed. Additionally, the IgG4-positive cell count significantly surpassed the 10/high power field, meeting the diagnostic criteria for IgG4-RD (Fig. 4).

The postoperative course was uneventful, and the patient was discharged on the third postoperative day. The patient underwent follow-up on an outpatient basis without additional medications since there were no signs of residual disease. The patient has been recurrence-free for six months following surgery and has not complained of symptoms related to the sympathectomy.

## Discussion

When lesions associated with IgG4-RD manifest across multiple organs, systemic steroid therapy is the treatment of choice [1]. However, when the lesions are confined to a single resectable organ, as in this case, the optimal treatment intervention is debatable. After confirming a IgG4-RD diagnosis, physicians may consider follow-up observation in asymptomatic cases and pharmaceutical intervention upon detection of lesion enlargement. However, there are notable disadvantages to both this approach and the administration of steroids. One, there is a lack of consensus regarding the side effects and duration of steroid therapy. Two, surgery may become more difficult to perform if lesion enlargement occurs during the follow-up period; however, despite the risk of flare-ups associated with systemic disease in the long term [2], the ability to follow up without resorting to steroid treatment is considered greatly advantageous for surgical resection.

Four cases of IgG4-RD occurring exclusively in the posterior mediastinum have been reported in previous literature (Table 1). In all cases, percutaneous biopsy was difficult, and in two cases, resection was performed as the intraoperative rapid pathological examination did not rule out malignancy [3, 4]. Likewise, in two cases, biopsy was performed only for diagnostic purposes [5, 6]. Clinical features of paravertebral lesions encompass challenges in performing percutaneous biopsy and a lack of subjective symptoms. Furthermore, it is difficult to distinguish them from lymphoma or other malignant diseases using biopsy alone. If there is a tendency toward enlargement, resection for diagnostic purposes is desirable. Consequently, if resection is necessary, paravertebral lesions require dissection tangential to the chest wall plane. The usefulness of robotic surgery for this purpose has been previously reported [7]. In this case, the tumor was relatively hard and widely tangential to the chest wall, and the application of an articulated forceps, a feature of robotic surgery, proved instrumental in achieving complete resection of the tumor.

**Table 1:** Reported cases of paravertebral tumor alone caused by IgG4-related disease

Author	Age (years)/sex	Symptom	Tumor size(cm)	Surgery	postoperative course
Uchida, et al. 2)	84/Female	None	5.6	VATS resection additional resection of azygos vein	No medication no recurrence 31 months after surgery
Hosaka, et al. 3)	62/Female	Cough,sputum	3.3	VATS resection additional resection of Part of the vertebral body	No medication no recurrence 11 months after surgery
Hirai, et al. 4)	71/Male	None	15	VATS biopsy only	No medication (observation period not stated)
Ozasa, et al. 5)	67/Male	None	None stated	VATS biopsy only	No medication no increase in size 7 months after surgery
Our case	58/Female	None	5.5	RATS resection	No medication no recurrence 12 months after surgery

### Electronic supplementary material

Below is the link to the electronic supplementary material.

Supplementary material 1

## Data Availability

Not applicable.

## Abbreviations

GCs: Glucocorticoids
ICS: Intercostal spaces
Ig: Immunoglobulin
IgG4-RD: Immunoglobulin G4-related disease
MRI: Magnetic resonance imaging
